# Understanding preparedness for extreme weather events among adults with chronic diseases: the interplay of illness perceptions, threat appraisal, and weather-related worry

**DOI:** 10.3389/fpubh.2026.1852635

**Published:** 2026-08-19

**Authors:** Yaira Hamama-Raz, Shiri Shinan-Altman

**Affiliations:** 1 School of Social Work, Ariel University, Ariel, Israel; 2 School of Social Work, Bar-Ilan University, Ramat Gan, Israel

**Keywords:** chronic illness, climate preparedness, extreme weather, illness perceptions, threat appraisal, weather-related worry

## Abstract

**Background:**

Extreme weather events are an increasing public health concern, particularly for adults living with chronic diseases. In this study, we examined whether illness perceptions were associated with preparedness for extreme weather events through a theoretically informed serial indirect association involving threat appraisal and weather-related worry, and whether these associations differed across respiratory disease, cancer, and cardiovascular disease groups.

**Methods:**

A cross-sectional study was conducted among 450 Israeli adults aged 50 years or older with a physician-diagnosed respiratory disease, cancer, or cardiovascular disease. Participants completed self-report measures of illness perceptions, threat appraisal, weather-related worry, and preparedness for extreme weather events.

**Results:**

More negative illness perceptions showed a significant serial indirect association with greater preparedness for extreme weather events through higher threat appraisal and greater weather-related worry. This indirect association was significant for both “intuitive” and “planned” preparedness behaviors across all three disease groups. The pattern of indirect associations specified in the serial mediation model was similar across groups. Of the nine interaction terms tested, one was significant, where disease group moderated the association between illness perceptions and weather-related worry. This association was significant among participants with respiratory disease, but not among those with cancer or cardiovascular disease. This exploratory finding should be examined in future research.

**Conclusions:**

This study found that illness perceptions, threat appraisal, weather-related worry, and preparedness for extreme weather events were interrelated among adults with chronic diseases, with a similar pattern of indirect associations across respiratory disease, cancer, and cardiovascular disease groups. Given the cross-sectional design, the proposed ordering of these variables should be interpreted as theoretically informed rather than as evidence of a temporal or causal pathway. These findings contribute to research at the intersection of illness self-regulation and climate-health adaptation and may inform future longitudinal research and preparedness interventions for medically vulnerable populations.

## Introduction

Extreme weather events, particularly heatwaves and cold spells, are a growing public health concern (1). Climate change is increasing the frequency, duration, and intensity of extreme weather events and amplifying weather-related threats to health, while temperature extremes are putting growing pressure on both population health and health systems (2, 3). Evidence further indicates that individuals living with chronic diseases are at elevated risk when exposed to both high and low ambient temperatures. In particular, temperature extremes have been associated with increased cardiovascular and respiratory morbidity and mortality (4, 5). These findings underscore the importance of preparedness for extreme weather events as a public health priority. At the public health level, preparedness for extreme weather events has emerged as a central component of climate-resilient health systems and disaster risk reduction strategies (7). At the individual level, preparedness refers to the behavioral and cognitive efforts undertaken by individuals in anticipation of, and in response to, extreme weather events, encompassing actions such as securing medications, adjusting daily routines, monitoring weather warnings, and planning for disruptions to healthcare access (6, 7). These findings underscore the importance of preparedness for extreme weather events as a public health priority.

Preparedness may be especially important among this cohort, namely, individuals living with chronic illnesses, as their health is more vulnerable to environmental stressors and disruptions in care. Specifically, respiratory diseases are directly affected by temperature extremes through airway inflammation and exacerbation of conditions such as asthma and chronic obstructive pulmonary disease (COPD) (7). Cardiovascular diseases are similarly vulnerable, as heat and cold exposure impose additional strain on the cardiovascular system, increasing the risk of acute cardiac events (7). Cancer patients face heightened vulnerability due to immunosuppressive treatments, dehydration related to cancer therapies, and dependence on continuous healthcare services, all of which may be disrupted during extreme weather events (8). In the present study, we focused on respiratory disease, cardiovascular disease, and cancer, given that these conditions represent major categories of non-communicable diseases and are especially vulnerable to the health impacts of extreme weather events (9, 10). Accordingly, individuals living with these chronic illnesses may have a particularly strong need to be prepared for the effects of extreme weather events.

However, objective medical vulnerability alone may not fully explain why some individuals engage in preparing for extreme weather events, whereas others do not. According to Leventhal's Common-Sense Model (CSM) of illness self-regulation, individuals actively construct cognitive and emotional representations of their illness in order to make sense of their health situation and guide their coping behavior (11). These representations are organized around five key dimensions: identity (the symptoms and label associated with the illness), timeline (beliefs about its duration and trajectory), consequences (expected impact on daily functioning and quality of life), controllability (beliefs about whether the illness can be managed or cured), and coherence (the degree to which the illness makes sense to the individual) (11). Emotional representations, such as fear or distress in response to the illness, constitute an additional dimension that operates in parallel with cognitive appraisals (11). Illness perceptions are therefore likely to influence whether extreme weather events are interpreted as personally relevant and health-threatening. Empirical studies have consistently demonstrated that more negative illness perceptions such as perceiving greater consequences or lower controllability, are associated with poorer health outcomes, reduced self-management, and maladaptive coping across a range of chronic conditions (11, 12). From this perspective, preparedness may depend not only on individuals' medical condition, but also on the meaning they attribute to their illness and its potential interaction with environmental threat.

Complementing the CSM, Protection Motivation Theory (PMT) (13) provides a useful framework for understanding how illness perceptions may be associated with protective action. PMT posits that protective behavior is motivated by two parallel appraisal processes: threat appraisal, which involves evaluating the severity of a potential harm and one's personal vulnerability to it, and coping appraisal, which involves evaluating one's capacity to effectively respond to the threat (13, 14). According to PMT, protective responses are shaped by these appraisal processes, such that perceiving a threat as serious and personally relevant increases the motivation for self-protection (14). Evidence from health-behavior research further supports the role of appraisal processes in predicting protective behavior (15). In the present context, threat appraisal may be especially important given that extreme weather events may be construed as a direct threat to health among individuals living with chronic diseases (16). Threat-related appraisals are also often accompanied by emotional responses. In particular, worry is conceptualized as an affective response to perceived risk that is distinct from cognitive threat appraisal and reflects an emotional engagement with potential harm (17, 18). In environmental-risk research, worry has emerged as a particularly salient response and has been associated with greater engagement in protective and pro-environmental behavior (17, 18). Accordingly, threat appraisal and worry may represent theoretically relevant psychological processes that are statistically associated with the link between illness perceptions and preparedness for extreme weather events.

However, despite this theoretical rationale, important gaps remain in understanding preparedness for extreme weather events among individuals living with chronic illnesses. Existing research has focused primarily on risk perception and coping, with comparatively little attention to preparedness itself (6). In addition, prior climate-health research involving chronic diseases has largely compared individuals with and without chronic diseases, showing heightened climate-related awareness, risk appraisal, and weather-related worry among those living with chronic illnesses, rather than examining potential differences across specific illness groups (16). As a result, it remains unclear whether the psychological processes linking illness perceptions with preparedness operate similarly or differently across respiratory disease, cardiovascular disease, and cancer. In the present study we therefore examined preparedness for extreme weather events among adults aged 50 years or older living with one of these chronic diseases. Drawing on the CSM and PMT, we hypothesized as follows:

**H1**. More negative illness perceptions would be associated with greater preparedness for extreme weather events within a theoretically informed serial indirect-association model, such that more negative illness perceptions would be associated with greater threat appraisal, greater threat appraisal would be associated with higher weather-related worry, and higher weather-related worry would be associated with greater preparedness.
**H2**. Chronic illness type (i.e., respiratory disease, cancer, or cardiovascular disease) would moderate the associations among illness perceptions, threat appraisal, weather-related worry, and preparedness for extreme weather events. (It should be noted that because the existing literature suggests distinct but not directly comparable vulnerability profiles across these disease groups, and because direct comparative evidence on these psychological pathways remains limited, no directional hypothesis was specified regarding the pattern of between-group differences).

## Methods

### Participants

An *a priori* power analysis was performed using G^*^Power 3 (19). For a one-way analysis of variance with three groups, α = 0.05, a small-to-moderate effect size (*f* = 0.15), and power of 0.80, the required sample size was 432 participants. A further calculation based on Kenny's (20) recommendations for mediation analysis indicated that, assuming β = 0.15 or higher for any direct effect in the model, α = 0.05, and power of 0.80, a sample of 450 participants would be required to detect an indirect effect.

The final study sample therefore comprised 450 Israeli adults aged 50 years or older with a physician-diagnosed chronic illness, including respiratory disease (*n* = 149), cancer (*n* = 151), and cardiovascular disease (*n* = 150). Although the overall sex distribution was nearly balanced (47.3% men and 52.8% women), the three illness groups differed substantially in this respect: women constituted the majority of the respiratory (66.4%) and cancer groups (69.5%), whereas men predominated in the cardiovascular group (78.0%) (*p* < 0.001). The mean age of the sample was 63.88 years (*SD* = 5.93; range = 50–88), with participants in the cardiovascular group (Mean = 66.95 years, SD = 8.77) being older on average than those in the other two groups (respiratory disease: Mean = 60.42 years, SD = 8.32, cancer: Mean = 64.25 years, SD = 8.52) (*p* < 0.001). Most participants were married or in a committed relationship (71.8%) and reported a mean of 2.67 children (*SD* = 1.48), with no significant differences across illness groups. Overall, the sample was relatively well educated: 55.6% of participants reported having a higher education, and the mean duration of education was 15.34 years (*SD* = 2.94), with no illness group difference. Self-reported economic status was most commonly described as average (45.1%), followed by above average (30.9%) and below average (24.0%), with no group difference. Employment status differed significantly across illness groups, with participants who had respiratory diseases showing the highest employment rate (73.2%), compared with those who had cancer (51%) or cardiovascular disease (46%) (*p* < 0.001). Most participants identified as secular (73.3%) and resided in urban areas (80.2%). In terms of self-rated health, slightly more than half of the sample (54.5%) rated their health as good or excellent, whereas the remainder rated it as fair (40.4%) or poor (5.1%), with no group difference. Illness duration varied significantly across illness groups (*p* < 0.001), being longest among participants with respiratory diseases (Mean = 31.46 years, SD = 19.64) and shortest among those with cancer (Mean = 7.50 years, SD = 6.76) (cardiovascular disease: Mean = 13.46 years, SD = 10.17).

### Measures

Participants were asked to complete a battery of self-report questionnaires that included the following measures: *sociodemographic and health-related characteristics*: participants provided sociodemographic information, including gender, age (in years), area of residence (urban/rural), marital status (married, divorced, widowed, single), number of children, years of education, highest level of education attained, employment status (full-time, part-time, self-employed, unemployed, pensioner, stay-at-home parent), and religiosity (secular, traditional but not very religious, traditional and religious/Orthodox). Self-rated health was assessed using a single item: “In general, how do you rate your health?” rated on a scale ranging from 1 (bad) to 4 (excellent) (21).

*Health information*: participants were first asked whether they had a chronic disease (yes/no). Those who responded affirmatively were then asked to specify the type of chronic disease. Only participants who reported a diagnosis of respiratory disease, cardiovascular disease, or cancer, without comorbidity across these disease categories, were included in the study. Participants were also asked to report the number of years since diagnosis.

*Illness perceptions* were assessed using the Brief Illness Perception Questionnaire [Brief IPQ; (22)]. Although the Brief IPQ consists of nine items, only the eight scaled items were used in the present study; the ninth item, which assesses perceived causes of the illness, was not included. The eight items represent three domains: cognitive representations (five items consisting of consequences, timeline, personal control, treatment control, and identity), emotional representations (two items consisting of concern and emotional response), and illness comprehensibility/coherence (one item). These items are rated on a scale from 0 to 10, and a mean score ranging from 0 to 10 was calculated, with higher scores indicating more negative illness perceptions. In the current study, Cronbach's alpha for the eight-item scale was α = 0.78.

*Climate change-related worry in the context of extreme weather events* was assessed using the Climate Change Worry Scale (23). This scale measures participants' worry about climate change and the potential contribution of climate change to extreme weather events. It includes 10 items (e.g., “I am worried that outbreaks of severe weather could be a result of climate change”) rated on a five-point Likert scale ranging from 1 (never) to 5 (often). The scale was translated from English into Hebrew using a back-translation procedure conducted by a professional English translator. This Hebrew version has been used in several previous studies conducted in Israel (16, 24). For the present study, the Hebrew version was contextually adapted to the Israeli setting. Participants were informed that the study concerned extreme weather changes in Israel, particularly heatwaves and cold spells, and were asked to respond to the items within this conceptual framework. Accordingly, the scale was used to assess climate change-related worry as framed in relation to extreme weather events, rather than worry about climate change in a purely global or abstract sense. For brevity, this construct is referred to hereafter as weather-related worry. An average score was calculated, with higher scores indicating greater weather-related worry. In the present study, Cronbach's alpha was α = 0.94.

*Health-related threat appraisal of extreme weather events* was measured using an adapted version of three items assessing threat perceptions of climate change (25), which were modified linguistically and contextually to refer specifically to extreme weather events and their perceived health implications in the Israeli context. Participants rated their agreement with each statement (e.g., “I feel that my health is threatened by extreme weather events”) on a five-point Likert scale ranging from 1 (strongly disagree) to 5 (strongly agree). Before completing the questionnaire, participants were informed that the study concerned extreme weather changes in Israel, particularly heatwaves and cold spells, and were asked to respond to the items within this conceptual framework. For brevity, this construct is referred to hereafter as threat appraisal. An average score was computed, with higher scores indicating greater threat appraisal. In the current study, Cronbach's alpha was α = 0.65.

*Preparedness for extreme weather events* was assessed using a nine-item self-report measure adapted for the context of extreme weather events, drawing on prior work on climate change preparedness and resilience, including the ClimateMind50+ questionnaire and the Climate-Ready Boston Progress Report (26, 27). In the present study, the measure was contextually adapted to the Israeli setting and focused primarily on preparedness behaviors related to locally relevant extreme weather conditions, particularly heatwaves, cold spells, high air pollution, and related disruptions. The measure was initially examined in a preliminary pilot study conducted among 200 adults with chronic illnesses and 200 healthy adults. In that study, two components of preparedness were identified via exploratory factor analysis: *intuitive behaviors* (five items; e.g., “I avoid leaving the house during days of extreme heat or high air pollution”), referring to deliberate actions based on one's own initiative, and *planned behaviors* (four items; e.g., “I talk to my doctor or health professional about the effects of weather on my health”), referring to deliberate actions that require advance planning and may involve other people. In the pilot study, the “intuitive behaviors” component had an eigenvalue of 2.86, explained 34.4% of the variance, showed factor loadings ranging from 0.69 to 0.79, and demonstrated good internal consistency (Cronbach's α = 0.79). The “planned behaviors” component had an eigenvalue of 2.44, explained 23.3% of the variance, showed factor loadings ranging from 0.66 to 0.81, and demonstrated acceptable internal consistency (Cronbach's α = 0.73). *In the present study*, the same two-factor structure was observed. The “intuitive behaviors” factor had an eigenvalue of 3.25, explained 43.9% of the variance, showed factor loadings ranging from 0.61 to 0.76, and demonstrated good internal consistency (Cronbach's α = 0.80). The “planned behaviors” factor had an eigenvalue of 2.95, explained 15.62% of the variance, showed factor loadings ranging from 0.60 to 0.81, and demonstrated acceptable internal consistency (Cronbach's α = 0.76). Mean scores were calculated for each subscale, with higher scores indicating greater preparedness for extreme weather events.

### Procedure

The study was conducted in accordance with internationally recognized ethical standards for research involving human participants. Approval was obtained from the institutional review board of the first author's institution (IRB No. AU-SOCYHR-20251130). Participants were recruited through iPanel, Israel's largest probability-based online research panel, which adheres to the ESOMAR international code of ethical practice and participant protection. This nationally representative panel includes more than 100,000 adults aged 18–85 years who have previously consented to participate in online survey studies (https://www.ipanel.co.il/). All participants received detailed information about the study objectives and procedures before enrollment and provided informed consent electronically. Data collection took place in February 2026. The inclusion criteria were being 50 years of age or older, having a physician-diagnosed chronic illness (i.e., respiratory disease, cancer, or cardiovascular disease), being able to read and understand Hebrew, and providing informed consent prior to participation.

### Data analysis

Data were analyzed using SPSS version 31. Descriptive statistics were calculated for participants' demographic characteristics and the study variables. Normality of the study variables was assessed using skewness and kurtosis indices, and no substantial deviations from normality were detected (skewness values ranged from −0.38 to 0.96, SE = 0.12; kurtosis values ranged from −0.10 to 0.86, SE = 0.23). Common method bias was examined using Harman's single-factor test (28). The single factor accounted for 32.22% of the total variance. Harman's test is only a preliminary diagnostic for this same-source, same-time survey design, and thus common method bias may still pose a threat to the validity of the findings. Analyses of variance (ANOVAs) and Scheffé *post hoc* comparisons were conducted to examine group differences in the study variables, as well as in the dimensions of illness perception. Pearson correlations and *t*-tests were conducted to examine the associations between demographic characteristics and preparedness for extreme weather events, in order to identify the demographic characteristics to be controlled for. Furthermore, because the study model included multiple predictors, multicollinearity diagnostics were conducted. VIF values ranged from 1.00 to 1.76, and tolerance values ranged from 0.79 to 0.99, both of which were well within acceptable thresholds, indicating no evidence of multicollinearity. The hypotheses were examined using Hayes' (29) PROCESS Model 92, a moderated serial mediation model. Because the data were cross-sectional, the model was interpreted as a theoretically informed conditional process model estimating indirect associations, rather than as evidence of causal or temporal mediation. Illness perceptions were defined as the independent variable, and preparedness for extreme weather events (intuitive and planned behaviors) as the dependent variable. Threat appraisal and weather-related worry were defined as the mediator variables, and illness group as the moderator, represented by two variables with orthogonal coding. Both hypotheses were examined using the total score of illness perceptions. Variables were standardized, and simple slopes were used to interpret significant interactions. Bootstrapping with 5,000 samples and 95% confidence intervals was used. The Bonferroni correction for multiple comparisons was applied.

## Results

### Descriptive results

Group differences in the study variables (Table 1) indicated that threat appraisal related to the effects of extreme weather conditions was higher among participants in the respiratory disease group than among those in the cancer or cardiovascular disease groups. No other significant differences were found between the disease groups.

**Table 1:** Means, standard deviations, and *F* values for the study variables by illness group (*N* = 450).

Study variables	Total *M* (*SD*) (*N* = 450)	Respiratory *M* (*SD*) (*n* = 149)	Cancer *M* (*SD*) (*n* = 151)	Cardio-vascular *M* (*SD*) (*n* = 150)	*F*(2, 447) (*p*) (η^2^)
Illness perceptions	4.60 (1.72)	4.54 (1.65)	4.45 (1.93)	4.81 (1.54)	1.82 (*p* = 0.163) (η^2^ = 0.008)
Threat appraisal	2.45 (0.92)	2.65 (0.93)	2.35 (0.92)	2.35 (0.88)	5.30 (*p* = 0.005) (η^2^ = 0.023)
Weather-related worry	2.08 (0.82)	2.15 (0.83)	1.98 (0.76)	2.11 (0.86)	1.72 (*p* = 0.181) (η^2^ = 0.008)
Preparedness- intuitive behaviors	3.42 (0.82)	3.51 (0.81)	3.33 (0.80)	3.43 (0.83)	1.81 (*p* = 0.165) (η^2^ = 0.008)
Preparedness- planned behaviors	1.93 (0.76)	2.03 (0.84)	1.85 (0.70)	1.92 (0.72)	2.07 (*p* = 0.127) (η^2^ = 0.009)

Range: illness perceptions 0–10; Threat appraisal, Weather-related worry, Preparedness 1–5. Bonferroni correction for multiple comparisons was applied, and the *p*-value was set at < 0.010 (0.05/5).

Significant positive correlations were found among all study variables (Table 2). More severe illness perceptions, higher threat appraisal, and greater weather-related worry were associated with higher preparedness for the effects of extreme weather events, reflected in both intuitive and planned behaviors. In addition, more severe illness perceptions, higher threat appraisal, and greater weather-related worry were positively associated with one another.

**Table 2:** Pearson correlations among the study variables (*N* = 450).

Study variables	1.	2.	3.	4.	5.
1.Illness perceptions	1				
2.Threat appraisal	0.42^*^	1			
3.Weather-related worry	0.33^*^	0.60^*^	1		
4.Preparedness- intuitive behaviors	0.24^*^	0.30^*^	0.44^*^	1	
5.Preparedness- planned behaviors	0.31^*^	0.44^*^	0.66^*^	0.52^*^	1

^*^p < 0.001. Bonferroni correction for multiple comparisons was applied, and the *p*-value was set at < 0.005 (0.05/10).

Correlations between preparedness for the effects of extreme weather events (intuitive and planned behaviors) and sociodemographic and health-related variables revealed a negative association with participants' number of children for intuitive behaviors (*r* = −0.13, *p* = 0.003). Preparedness was not associated with gender (*p* = 0.191 and *p* = 0.671, for intuitive and planned behaviors, respectively), age (*p* = 0.845 and *p* = 0.372), years of education (*p* = 0.889 and *p* = 0.645), economic status (*p* = 0.364 and *p* = 0.109), or illness duration (*p* = 0.684 and *p* = 0.954). Therefore, the hypotheses were tested while controlling for participants' number of children.

### Hypotheses testing

The two hypotheses were examined using Hayes' (29) PROCESS Model 92, a moderated serial mediation model. Illness perception was defined as the independent variable, threat appraisal and weather-related worry as the mediators, disease groups as the moderator (represented by two variables with orthogonal coding), and preparedness for the effects of extreme weather events (intuitive and planned behaviors) as the dependent variable. Participants' number of children was controlled for. Variables were standardized, and interaction terms were created by multiplying the two group variables by the independent and mediator variables.

**Figure 1:**
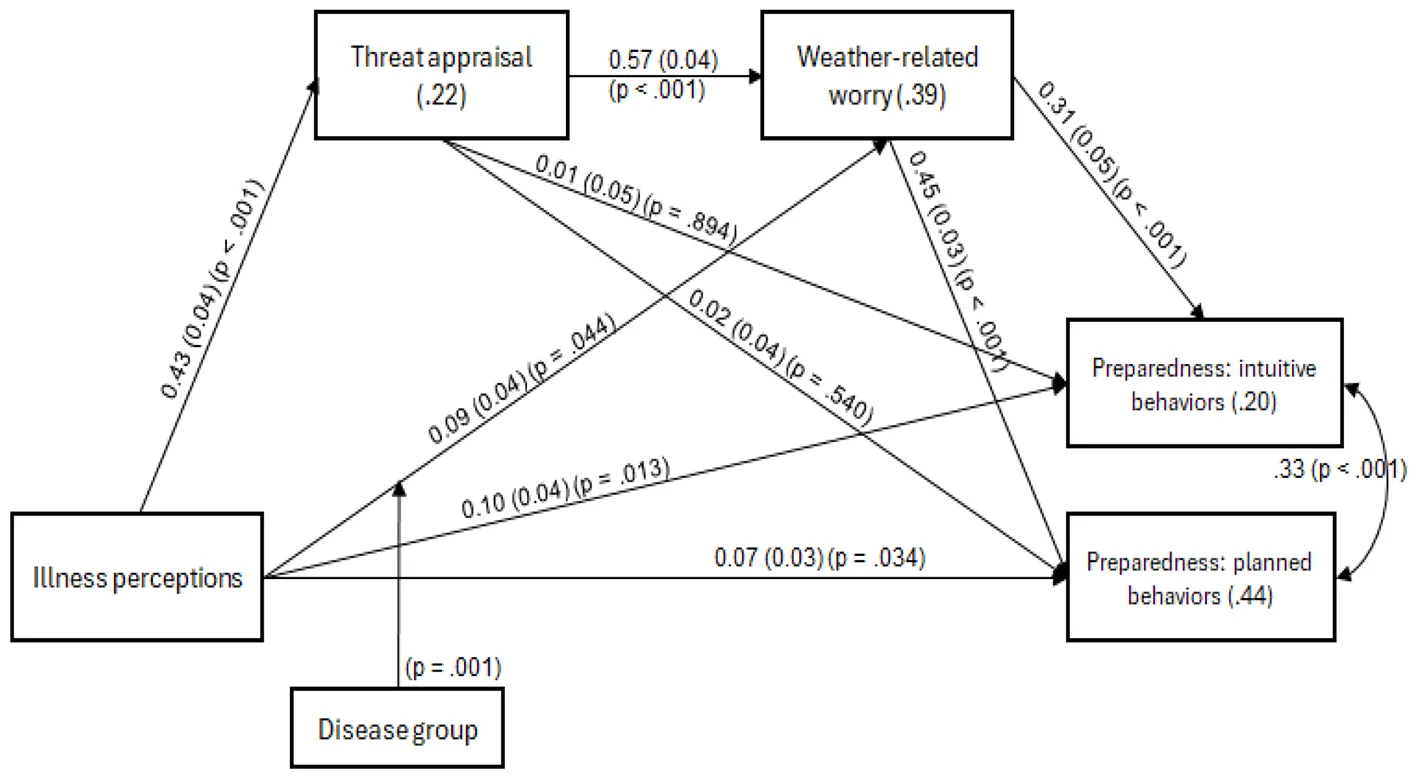
Moderated serial mediation for preparedness for extreme weather events, with illness perceptions, threat appraisal, and weather-related worry, and the moderating effect of disease group. Values on one-way arrows: unstandardized *B* (*SE*), values within rectangles: *R*^2^, value on a two-way arrow: Pearson *r*. For clarity, only significant moderation effects are shown (others are in Table 3). Bonferroni correction for multiple comparisons was applied, and the *p*-value was set at <0.005 (0.05/9).

Results of the moderated serial mediation model are presented in Figure 1 (Full coefficient values are presented in Supplementary Material Table 1). The findings indicated that, within the theoretically specified model, illness perception was positively associated with threat appraisal, threat appraisal was positively associated with weather-related worry, and weather-related worry was positively associated with preparedness for the effects of extreme weather events, including both intuitive and planned behaviors.

Accordingly, the full indirect effect was significant for both intuitive behaviors [respiratory disease: unstandardized effect = 0.05, SE = 0.02, 95% CI (0.02, 0.09); cancer: unstandardized effect = 0.06, SE = 0.02, 95% CI (0.02, 0.10); cardiovascular disease: unstandardized effect = 0.13, SE = 0.04, 95% CI (0.06, 0.21)] and planned behaviors [respiratory disease: unstandardized effect = 0.10, SE = 0.03, 95% CI (0.05, 0.17); cancer: unstandardized effect = 0.08, SE = 0.02, 95% CI (0.04, 0.13); cardiovascular disease: unstandardized effect = 0.14, SE = 0.04, 95% CI (0.08, 0.23)]. Because all interactions involving the full indirect association specified in the serial mediation model were non-significant (see Table 3), this pattern of associations was similar across all three disease groups. These findings support the hypothesized pattern of indirect associations; however, they do not establish the temporal ordering of the variables. Interpretation should also take into account the relatively low internal consistency of the threat appraisal measure. The first hypothesis was therefore supported at the level of statistical association.

**Table 3:** The moderating effects of illness group on the mediated associations between illness perceptions and preparedness (*N* = 450).

Dependent variable	Illness perceptions x illness group	Threat appraisal x illness group	Weather-related worry x illness group
Threat appraisal	*F*_ch_(2, 443) = 1.16, *p* = 0.314	—	—
Weather-related worry	*F*_ch_(2, 440) = 6.88, *p* = 0.001	*F*_ch_(2, 440) = 2.45, *p* = 0.088	—
Preparedness- intuitive behaviors	*F*_ch_(2, 437) = 0.47, *p* = 0.623	*F*_ch_(2, 437) = 0.09, *p* = 0.911	*F*_ch_(2, 437) = 0.20, *p* = 0.820
Preparedness- planned behaviors	*F*_ch_(2, 437) = 0.19, *p* = 0.829	*F*_ch_(2, 437) = 2.54, *p* = 0.080	*F*_ch_(2, 437) = 2.09, *p* = 0.125

Bonferroni correction for multiple comparisons was applied, and the *p*-value was set at < 0.005 (0.05/9).

Regarding the moderating role of the disease group, Table 3 presents all moderating effects. The results indicated that most interaction terms were not significant, with the exception of the interaction between illness perception and disease group in association with weather-related worry. It should be noted that only number of children was controlled for in the model, as it was the only variable significantly associated with the dependent variables. However, as the three disease groups differed significantly in age, sex distribution, and illness duration, the model was re-analyzed while controlling for them as well. Similar results were found, and the interaction between illness perception and disease group in association with weather-related worry, was significant (*p* = 0.001). Its interpretation revealed similar results to the ones below.

**Figure 2:**
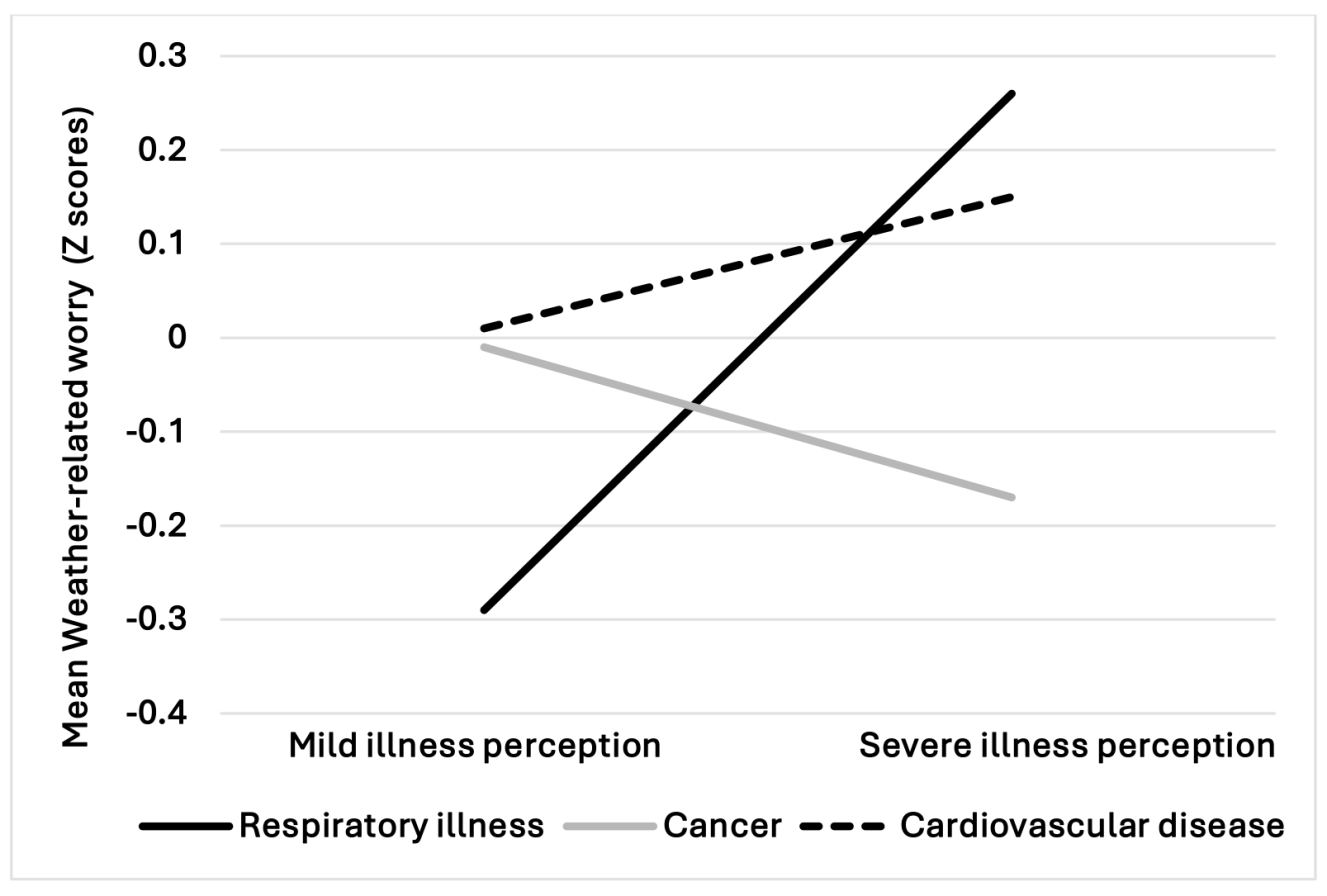
Illness perceptions and weather-related worry about the effects of extreme weather events by disease group.

Analysis of this significant interaction showed that the comparison between participants in the respiratory disease group and the other groups (cancer and cardiovascular disease) was significant [unstandardized *B* = −0.28, SE = 0.09, *p* = 0.002, 95% CI (−0.46, −0.11)], whereas the comparison between participants with cancer and those with cardiovascular disease was not significant [unstandardized *B* = 0.15, SE = 0.10, *p* = 0.149, 95% CI (−0.05, 0.35)]. Simple slopes analysis showed that the association between illness perception and worry about the effects of extreme weather events was positive and significant in the respiratory disease group [unstandardized effect = 0.28, SE = 0.07, *p* < 0.001, 95% CI (0.13, 0.42)], but was not significant in the cancer group [unstandardized effect = −0.08, SE = 0.06, *p* = 0.180, 95% CI (−0.21, 0.04)] or the cardiovascular disease group [unstandardized effect = 0.07, SE = 0.08, *p* = 0.426, 95% CI (−0.10, 0.23)] (Figure 2). In other words, more negative illness perceptions were associated with greater weather-related worry about the effects of extreme weather events among participants in the respiratory disease group, whereas such an association was not found among participants in the cancer or cardiovascular disease groups. The second hypothesis was therefore only partially supported.

As can be seen, the full serial mediation model was significant across all three disease groups. The model involving illness perception, threat appraisal, and preparedness was not significant for either intuitive or planned behaviors in any disease group, as the associations between threat appraisal and preparedness were not significant, nor were the relevant interaction terms [intuitive behaviors: respiratory disease: unstandardized effect = −0.02, SE = 0.03, 95% CI (−0.07, 0.04); cancer: unstandardized effect = 0.01, SE = 0.03, 95% CI (−0.06, 0.07); cardiovascular disease: unstandardized effect = −0.01, SE = 0.05, 95% CI (−0.11, 0.08); planned behaviors: respiratory disease: unstandardized effect = −0.02, SE = 0.02, 95% CI (−0.07, 0.03); cancer: unstandardized effect = 0.05, SE = 0.03, 95% CI (−0.01, 0.11); cardiovascular disease: unstandardized effect = −0.01, SE = 0.04, 95% CI (−0.09, 0.07)].

By contrast, the model involving illness perceptions, weather-related worry, and preparedness was significant for participants in the respiratory disease group, but not for participants in the cancer or cardiovascular disease groups. Specifically, in this model, the moderated mediation was significant for both intuitive and planned behaviors. The indirect effect was significant among participants in the respiratory disease group [intuitive behaviors: unstandardized effect = 0.08, SE = 0.03, 95% CI (0.03, 0.14); planned behaviors: unstandardized effect = 0.15, SE = 0.04, 95% CI (0.08, 0.25)], but was not significant in the other groups [intuitive behaviors: cancer: unstandardized effect = −0.02, SE = 0.02, 95% CI (−0.06, 0.01); cardiovascular disease: unstandardized effect = 0.02, SE = 0.03, 95% CI (−0.04, 0.09); planned behaviors: cancer: unstandardized effect = −0.03, SE = 0.02, 95% CI (−0.09, 0.01); cardiovascular disease: unstandardized effect = 0.03, SE = 0.04, 95% CI (−0.04, 0.10)].

## Discussion

In the present study, we examined preparedness for extreme weather events among adults with respiratory disease, cancer, and cardiovascular disease, focusing on the psychological mechanisms linking illness perceptions to preparedness and the possible moderating role of disease type. The findings supported the proposed serial mediation model whereby more negative illness perceptions were associated with greater preparedness through higher threat appraisal and greater weather-related worry, whereas disease type moderated only one specific pathway rather than the overall model.

Regarding the first hypothesis, the findings supported the expectation that more negative illness perceptions would be indirectly associated with greater preparedness for extreme weather events through greater threat appraisal and weather-related worry. This indirect association was significant for both intuitive and planned preparedness behaviors across all three disease groups. Although the opposite direction of mediation cannot be ruled out, these findings are consistent with the CSM, which proposes that cognitive and emotional representations of illness are relevant to coping and adaptation (12, 30). However, the cross-sectional design does not establish that illness perceptions preceded threat appraisal, weather-related worry, or preparedness. Thus, the proposed ordering should be understood as a theoretically informed analytic specification rather than an empirically demonstrated temporal sequence. Reverse or reciprocal associations are also plausible; for example, individuals who have already undertaken preparedness behaviors may subsequently become more attentive to weather-related health threats and report greater weather-related worry. This pattern is also broadly consistent with the PMT, according to which threat-related appraisals play a central role in motivating self-protective behavior (13).

Support for the first hypothesis is particularly meaningful as it makes a step toward extending existing research on illness perceptions beyond disease management to climate-related preparedness. Previous studies have consistently shown that cognitive and emotional representations of illness are linked to coping and adjustment across chronic conditions (12, 29). The current findings also align with emerging climate-health evidence indicating that adults and individuals with chronic illnesses tend to appraise greater risks, experience heightened worry, and express stronger preparedness needs when facing extreme weather events (6, 16, 31). These results suggest that illness perceptions may be associated with how people interpret and respond to environmental threats that could exacerbate their medical condition.

Another important aspect of support for the first hypothesis is that the proposed serial mediation model was significant for both intuitive and planned preparedness behaviors. Intuitive behaviors likely reflect immediate protective actions, whereas planned behaviors require greater forethought, coordination, and engagement with health or community support systems. This distinction is consistent with evidence that adults' heat adaptation includes both individual coping responses and more formalized preparedness strategies (6). The fact that the same psychological pathway was associated with both forms of preparedness suggests that illness perceptions, threat appraisal, and worry may be relevant across multiple types of protective behaviors.

The findings also provide information about the pattern of associations among the proposed mediators. Although illness perceptions were associated with threat appraisal, and threat appraisal was associated with weather-related worry, the indirect association involving threat appraisal alone was not significant once weather-related worry was included in the model. Within the proposed conceptual ordering, weather-related worry showed a stronger association with preparedness than threat appraisal. However, because temporal precedence cannot be determined from these data, weather-related worry should not be interpreted as a demonstrated mechanism through which perceived risk produces preparedness. This interpretation is consistent with research suggesting that climate-related worry and anxiety may serve as action-oriented responses that are associated with adaptive or protective behavior (32, 33).

With regard to the second hypothesis, the findings provided only very partial support for the expectation that chronic disease type would moderate the associations among illness perceptions, threat appraisal, weather-related worry, and preparedness. Contrary to expectations, most interaction terms were not significant, whereas the full serial mediation pathway was significant across all three groups: respiratory disease, cancer, and cardiovascular disease. These findings may suggest that the pattern of associations specified in the proposed model was largely similar across chronic disease groups. Although these diseases differ substantially in etiology, course, and treatment burden (34–36), the present findings propose that illness perceptions, threat appraisal, weather-related worry, and preparedness were associated in broadly comparable ways across the three groups. Longitudinal research is needed before concluding that a common self-regulatory process operates similarly across disease groups. However, the association between illness perceptions and weather-related worry tended to vary by disease type. Specifically, more negative illness perceptions were associated with greater worry about extreme weather events among participants with respiratory disease, but not among those with cancer or cardiovascular disease. Thus, although disease type did not alter the full serial mediation model, it tended to shape one specific emotional pathway within the model. This finding is exploratory, yet it is clinically plausible, as respiratory disease may make environmental threats especially immediate and tangible through breathing difficulties, symptom exacerbation, and functional discomfort (37, 38). Given the well-documented sensitivity of chronic respiratory conditions to heat, cold, and air-quality changes (39, 40), individuals with respiratory diseases who hold more negative illness perceptions may be particularly likely to experience extreme weather as a concrete and personally threatening source of worry. Further research is required to clarify this issue.

By contrast, the lack of a significant association between illness perceptions and weather-related worry in the cancer and cardiovascular disease groups should not be taken to indicate lower vulnerability. Both groups are highly vulnerable to extreme weather events, including increased cardiovascular morbidity and mortality and disruptions to cancer care and survivorship (41, 42). A more likely explanation for this lack of association is that, in these groups, weather-related worry is probably shaped by factors not captured in the present study, such as acute clinical history, treatment continuity concerns, trust in health systems, or perceived frailty. This possibility is consistent with the higher threat appraisal observed in the respiratory group, suggesting that disease type may influence how environmental threat is emotionally processed, even when the broader pattern remains similar across groups.

Taken together, the findings may have several practical implications, although these implications should be considered cautiously given the cross-sectional design. First, preparedness interventions for adults with chronic diseases may benefit from addressing patients' understanding of how their illness may interact with extreme weather events and what protective actions are feasible. Second, because weather-related worry was positively associated with preparedness in the proposed model, communication strategies may need to acknowledge realistic concerns while avoiding unnecessary distress. Future longitudinal and intervention studies are needed to determine whether modifying illness perceptions, threat appraisal, or weather-related worry contributes to improved preparedness behaviors. Third, the findings suggest that a broadly applicable preparedness framework may be used across chronic disease groups, while still allowing disease-specific tailoring, especially for individuals with respiratory diseases, whose illness perceptions may be more tightly coupled with emotional responses to weather threats. Recent public-health guidance for heat and health similarly emphasizes the importance of targeted communication and preparation for older adults and people with chronic diseases (43).

This study has several limitations that should be considered. First, the cross-sectional design precludes causal and temporal inferences. Although the serial mediation model was specified on the basis of the CSM, PMT, and prior literature, the directional ordering of illness perceptions, threat appraisal, weather-related worry, and preparedness represents an analytic assumption rather than a finding demonstrated by the present data. Thus, the observed indirect associations are consistent with the proposed model but do not establish it or rule out plausible alternative orderings. Reverse or reciprocal pathways cannot be excluded. For example, individuals who already engage in preparedness behaviors, such as monitoring warnings or consulting healthcare professionals, may subsequently appraise extreme weather events as more threatening and report greater worry. Longitudinal and prospective studies are needed to examine temporal precedence and compare alternative directional models. Second, the use of self-report measures may not fully reflect actual behavior during real extreme weather events. Third, all study variables referring to extreme weather events were examined within the Israeli context, where the study focused primarily on heatwaves and cold spells. Therefore, the findings should not be generalized to other types of extreme weather events, such as storms, floods, wildfires, or other weather-related emergencies that were not directly assessed in the present study. Fourth, the same-source, same-time survey design may have increased shared method variance, or common method bias, and may have inflated the observed indirect associations. Fifth, although the Bonferroni criterion for multiple comparisons was applied, the possibility of Type I error inflation cannot be ruled out. Sixth, although the preparedness measure showed a meaningful two-factor structure and acceptable internal consistency, further validation is needed. Similarly, the threat appraisal measure showed relatively low internal consistency, and its associations with the other study variables may have been attenuated by measurement error; therefore, future studies should further validate this measure. Seventh, only one of the nine interaction terms was significant, and this finding should therefore be interpreted as exploratory. Future research should continue to examine the role of disease group in the associations tested in the model. Eighth, participant recruitment through an online survey panel may have introduced selection bias toward more digitally literate and survey-experienced individuals, potentially underrepresenting individuals with lower digital access or health literacy. Finally, the sample was limited to Hebrew-speaking Israeli adults aged 50 years or older with one of three chronic diseases and no comorbidity across the target disease categories, which strengthens internal clarity but limits generalizability to more diverse and clinically complex populations.

Despite these limitations, the study contributes to a growing understanding of preparedness for extreme weather among people with chronic illnesses by highlighting the role of illness-related cognitions and emotions. The study also extends the CSM (11, 12) and PMT (13) to the weather-health context by showing their relevance to understanding preparedness for environmental threats, rather than only illness management or general health behavior. More broadly, the findings suggest that preparedness is shaped not only by medical vulnerability, but also by how individuals interpret their condition in relation to environmental threat. This perspective is especially relevant in the context of extreme weather events, where effective preparedness efforts should address both disease-related risk and the psychological processes that influence whether that risk is recognized and acted upon.

## Data Availability

The data analyzed in this study is subject to the following licenses/restrictions: the datasets analyzed during the current study are available from the corresponding author upon reasonable request. Requests to access these datasets should be directed to Yaira Hamama-Raz, yairahr@ariel.ac.il.
