# Supplementary material for: Understanding preparedness for extreme weather events among adults with chronic diseases: the interplay of illness perceptions, threat appraisal, and weather-related worry
Source: Front Public Health. 2026 Aug 19;14:1852635. doi: 10.3389/fpubh.2026.1852635 (PMC13533892; doi:10.3389/fpubh.2026.1852635)
Supplement: Supplementary file 1 [file Data_Sheet_1.docx]

**Supplementary material**

**Table 1**. Path coefficients for the moderated serial mediation model (*N* = 450)

| Path | *B* (*SE*) | *p* | 95%*CI* |
| --- | --- | --- | --- |
| Illness perceptions → threat appraisal | 0.43 (0.04) | < .001 | 0.34, 0.51 |
| Illness perceptions → weather-related worry | 0.09 (0.04) | .044 | 0.01 0.17 |
| Threat appraisal → weather-related worry | 0.57 (0.04) | < .001 | 0.48, 0.65 |
| Illness perceptions → preparedness: intuitive behaviors | 0.10 (0.04) | .013 | 0.02, 0.18 |
| Threat appraisal → preparedness: intuitive behaviors | 0.01 (0.05) | .894 | -0.09, 0.10 |
| Weather-related worry → preparedness: intuitive behaviors | 0.31 (0.05) | < .001 | 0.22, 0.39 |
| Illness perceptions → preparedness: planned behaviors | 0.07 (0.03) | .034 | 0.01, 0.13 |
| Threat appraisal → preparedness: planned behaviors | 0.02 (0.04) | .540 | -0.05, 0.09 |
| Weather-related worry → preparedness: planned behaviors | 0.45 (0.03) | < .001 | 0.38, 0.52 |

*Note.* Bonferroni correction for multiple comparisons was applied, and the p-value was set at < .005 (.05 / 9).
